# Three-dimensional imaging of vortex structure in a ferroelectric nanoparticle driven by an electric field

**DOI:** 10.1038/s41467-017-00318-9

**Published:** 2017-08-17

**Authors:** D. Karpov, Z. Liu, T. dos Santos Rolo, R. Harder, P. V. Balachandran, D. Xue, T. Lookman, E. Fohtung

**Affiliations:** 1 Department of Physics, New Mexico State University, Las Cruces, NM 88003 USA; 2 Department of General Physics, Physical-Technical Institute, National Research Tomsk Polytechnic University, Tomsk, 634050 Russia; 3 Condensed Matter Science and Technology Institute, School of Science, Harbin Institute of Technology, Harbin, 150080 China; 4 Los Alamos National Laboratory, Los Alamos, NM 87545 USA; 5 Institute for Photon Science and Synchrotron Radiation, Karlsruhe Institute of Technology, 76344 Eggenstein-Leopoldshafen, Germany; 6 Advanced Photon Source, Argonne National Laboratory, Argonne, IL 60439 USA; 7 State Key Laboratory for Mechanical Behavior of Materials, Xi’an Jiaotong University, Xian, 710049 China

## Abstract

Topological defects of spontaneous polarization are extensively studied as templates for unique physical phenomena and in the design of reconfigurable electronic devices. Experimental investigations of the complex topologies of polarization have been limited to surface phenomena, which has restricted the probing of the dynamic volumetric domain morphology in operando. Here, we utilize Bragg coherent diffractive imaging of a single BaTiO_3_ nanoparticle in a composite polymer/ferroelectric capacitor to study the behavior of a three-dimensional vortex formed due to competing interactions involving ferroelectric domains. Our investigation of the structural phase transitions under the influence of an external electric field shows a mobile vortex core exhibiting a reversible hysteretic transformation path. We also study the toroidal moment of the vortex under the action of the field. Our results open avenues for the study of the structure and evolution of polar vortices and other topological structures in operando in functional materials under cross field configurations.

## Abstract (web summary)

Imaging of topological states of matter such as vortex configurations has generally been limited to 2D surface effects. Here Karpov et al. study the volumetric structure and dynamics of a vortex core mediated by electric-field induced structural phase transition in a ferroelectric BaTiO_3_ nanoparticle.

## Introduction

Ferroelectric (FE) materials will likely have a broad range of applications in the next generation of electronics, such as non-volatile random access memorie (NRAM)^1–5^, and energy-storage and battery related technologies^6–8^. Nanoferroelectrics, unlike their bulk counterparts, show complex topological polarization textures^9^, including multi-stable states such as polar vortices^10^, which are formed due to long-range interactions mediated by elasticity and electrical boundary conditions. The influence of shape and size on vortex behavior has been extensively studied theoretically using electronic structure calculations and phase-field simulations^9–14^. Compared to bulk, nanoferroelectrics show promise of increasing the storage density of NRAMs 10,000-fold^1^. However, this expected benefit greatly relies on the possibility of observing and controlling structural transitions, including metastable states that allow for ease of switching of domains.

While the two-dimensional (2D) vortex domains in nanoferoelectrics have been extensively studied theoretically^9–14^, experimental effort has been directed toward synthesizing and observing polar structures in BaTiO_3_ (BTO) wires^15^, dots^16^, rods^17^, nanotubes^18^, as well as in Pb(Zr, Ti)O_3_ thin films and heterostructures^19^, and nanoparticles^20^. High-resolution transmission electron microscopy^21–23^ (HR-TEM), atomic force microscopy^24^, as well as numerous optical based experimental techniques^25^ have been successfully utilized to probe 2D vortex structures^26^. Combining HR-TEM with synchrotron radiation-based reciprocal space mapping has recently permitted the observation of long-range 2D vortex distributions in perovskite superlattices^27^. However, these approaches have not been transferred to direct non-invasive whole-volume studies of vortex structure due to fundamental limitations of the methods, forcing restrictions on studies in the presence of external perturbations, such as applied electric or magnetic fields, and leaving a gap in our understanding of dynamical processes.

Here, we use Bragg Coherent Diffraction Imaging (BCDI) to image the transformation path of the core of an individual vortex structure within a single BTO FE nanoparticle and elucidate the complex topologies of polarizations^9^, FE displacements, structural phases and mechanical responses of FE nanoparticles to electric fields. BCDI takes advantage of improvements in coherence at third and fourth generation light sources. Advancements in phase-retrieval algorithms enable the visualization of whole-volume information of the electron density distribution, atomic and ionic displacement fields within individual nanoparticles with nanoscale resolution^28^. For a review of advances in imaging of local structure using light sources, see refs ^29–34^.

## Results

### Electric field control of ferroelectric polarization

**Fig. 1:**
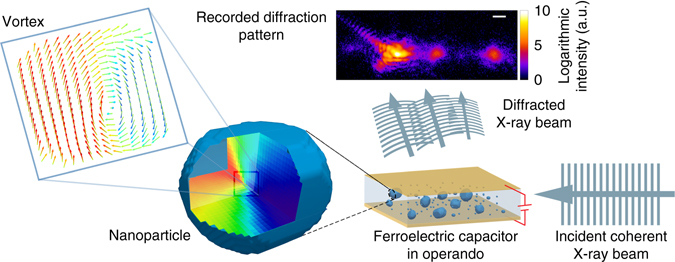
Experimental scheme of Bragg coherent diffraction imaging. Incident coherent X-ray beam is scattered by a nanoparticle embedded in conducting non-polarizing polymer with attached electrodes. Constructive interference patterns are recorded during application of an external electric field on the particle. Recorded high-resolution Bragg-peak diffraction carries information on the electron density and atomic displacement variations, allowing to reconstruct the complex process of defect evolution and monitoring of vortex. *Scale bar* corresponds to 0.1 Å^−1^

**Fig. 2:**
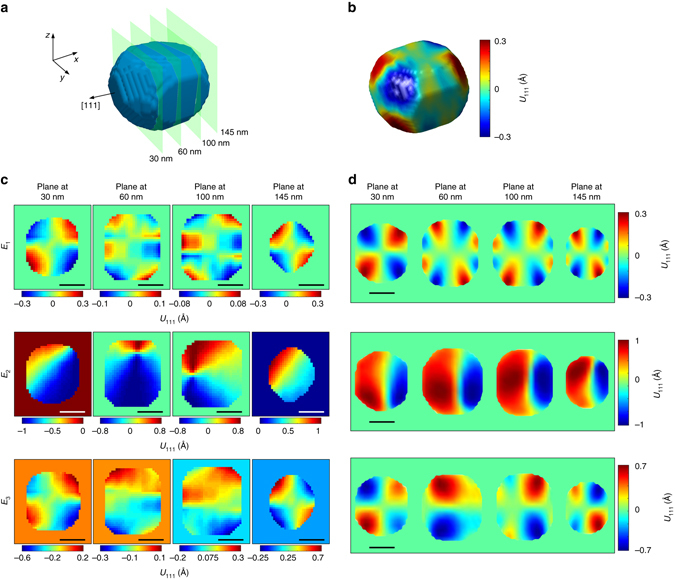
Correlations between Bragg coherent diffraction measurements and phase-field simulations. **a** A *blue* isosurface shows the reconstructed particle shape (amplitude) with *green planes* marking the locations of 2D cuts through the volume for the extracted planes in **d**. **b** Under zero electric field (initial state), the 3D projection of displacement field along the [111] direction is mapped onto the surface of the particle. **c** Slices through the particle volume at cut planes of 30, 60, 100, and 145 nm showing the inhomogeneity of the displacement and dynamics under external electric field. **d** Phase-field simulations for similar cut planes of 30, 60, 100, and 145 nm support the interpretation of experimental results. *Scale bars* correspond to 60 nm

To study a single FE nanoparticle morphology and its vortex structure, we designed a composite consisting of BTO nanoparticles and carbon nanoparticles dispersed within a non-ferroelectric polymeric dielectric matrix (Fig. 1, Supplementary Fig. 1 and the Methods section). Laboratory X-ray powder diffraction (Supplementary Fig. 2) confirms the crystalline nature of the BTO nanoparticles. In BCDI experiments the sample is illuminated with focused coherent X-ray beam (Fig. 1). A random orientation of the BTO nanoparticles along with the experimental geometry allows us to isolate and record the (111) Bragg reflections from a single BTO particle on an area detector. By applying an electric field in cycles and monitoring changes in diffraction pattern, we can differentiate BTO nanoparticles acting as nanocapacitor (Supplementary Fig. 3) from the particles which are electrically insulated in dielectric matrix (Supplementary Fig. 4). By iteratively inverting the coherent X-ray diffraction data, we obtain information on the three-dimensional (3D) Bragg-electron density distribution along with ionic displacement fields (Fig. 2b) with 20 nm spatial resolution (Supplementary Fig. 5). Supplementary Note 1 for more information.

**Fig. 3:**
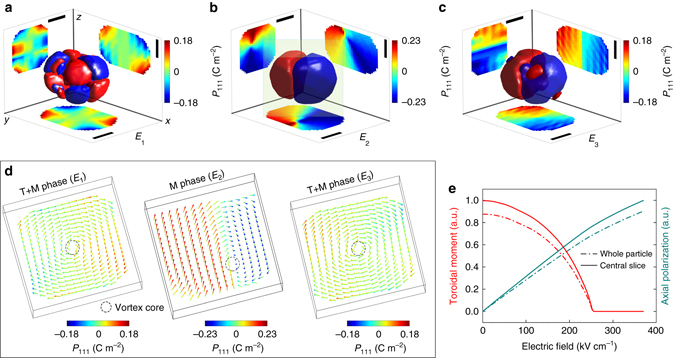
Three-dimensional nanodomain and vortex dynamics in BTO nanoparticle. Isosurface of the spontaneously formed nanodomain arrangements in BTO nanoparticle as obtained from Bragg Coherent Diffraction Imaging. Evolution of the spontaneous polarization distribution **a** at field *E*_1_ (0 kV cm^−1^), **b** at field *E*_2_ (223 kV cm^−1^), and **c** at field *E*_3_ (0 kV cm^−1^) shows that the in-plane components of **P**_111_ are always arranged in a flux-closure (vortex) manner in the virgin state. **d** Phase-field simulations confirm a mixture of Tetragonal and Monoclinic (T+M) structural phases that accounts for the vortex (clockwise) structure at zero electric field *E*_1_ (0 kV cm^−1^). **e** The curl of the axial polarization is characterized by an electric toroidal moment (${\bf{T}} = 1/{T_0}{\int} {\left( {{\bf{r}} \times {\bf{p}}} \right)} \,{\rm{d}}{V_{{\rm{cell}}}}$, where *T*_0_ is the toroidal moment without field at *E*_1_, *V*_cell_ is the volume of the cell located at position **r**, and **p** is the local dipole) from phase-field simulations. The disappearance of the toroidal moment indicates a vortex-to-polarization transformation in the nanoparticle^37^. Under field *E*_2_ (223 kV cm^−1^), the vortex core is off-centered with a predominant Monoclinic phase. As we decrease the field to remnant *E*_3_ (0 kV cm^−1^), the vortex core returns to the center of the particle. *Scale bars* correspond to 60 nm

Figure 2a shows the isosurface rendering of the particle’s shape, and Fig. 2b shows the [111] projection of the 3D displacement field **u**_111_(**r**) onto the isosurface at the virgin electric field state *E*_1_ = 0 kV cm^−1^. We further compare reconstructions of ionic displacements with phase-field simulations within the nanoparticle when exposed to 223 kV cm^−1^ (*E*_2_) and at remnant (*E*_3_ *= *0 kV cm^−1^) (Fig. 2d) states. The electric field magnitude estimates are obtained from the phase-field simulation based on the convergence of the model to the experimental results. The total elastic strain *ϵ*_*ij*_ can be estimated from **u**_111_(**r**) by *ϵ*_*ij*_ = 1/2 (∂**u** ^*i*^_111_ */*∂*x*_*j*_ + ∂**u** ^*j*^_111_ */*∂*x*_*i*_). The symmetric nature of the strain relative to the bulk structure enables coupling to the polarization and is given by

1 $$\epsilon _{ij}^o{\rm{ = }}{Q_{ijkl}}{P_k}{P_l}$$

where *Q*_*ijkl*_ is the electrostrictive tensor, *ϵ* ^o^_*ij*_ is the spontaneous strain, and the projections of the ferroelectric polarization along the *x*, *y*, and *z* directions are given by *P*_*k*_ and *P*_*l*_. The displacement field in Fig. 2b is indicative of a topological polar vortex in the nanoparticle given as isosurfaces in Fig. 3a–c. The electrostrictive coefficients can therefore be obtained as a fitting parameter by using Equation 1. The elastic coefficients used in the fit are consistent with phase-field simulations (Supplementary Note 2 and Supplementary Table 1). Since the displacements **u**_111_(**r**) are relatively small compared to the particle size, we assume that **u**_111_(**r**) scales with the local polarization, which can be obtained by a summation of Born effective charges^35, 36^. The formation of the vortex in the nanoparticle is a result of the competition between the elastic energy, the electrostatic energy, the gradient energy and boundary conditions, including nanoparticle shape, and surface facets (Supplementary Fig. 6). The elastic energy, which arises from the fact that BaTiO_3_ nanoparticle is constrained by the non-ferroelectric polymer matrix, drives the nanoparticle to adopt to a mixture of in-plane *P*_*x*_, *P*_*y*_ and out-of-plane *P*_*z*_ polarizations. The second major contribution is the electrostatic energy induced by the built-in electric fields. If the interface between the ferroelectric nanoparticle and the non-ferroelectric matrix is charge free or has a very small charge density, that is, $\nabla \cdot {\bf{P}} \approx 0$, the polarizations tend to align parallel to the interface. Finally, the gradient energy tends to change the direction and magnitude of the polarization. The three energy contributions lead to the topological structure observed in the nanoparticle (Fig. 3a).

### Structural phase transition and vortex core transformation path

An external electric field *E* in the [111] direction was applied to study the influence of *E* on the domain structure. The recorded Bragg coherent diffraction (Fig. 1 and Supplementary Fig. 3) shows evidence of structural phase coexistence within the nanoparticle and its transformation under fields *E*_1_ = 0 kV cm^−1^, *E*_2_ = 223 kV cm^−1^, and *E*_3_ = 0 kV cm^−1^. Slices of the reconstructed displacements (Fig. 2d) and polarization (Fig. 3a–c) at different depths within the nanoparticle for a given values of *E* show a heterogeneous distribution of the domains. At the intersection of the domain walls, we observe the formation of a polar vortex (Fig. 3d). The transformation path of the vortex^37^ is accompanied by a structural phase transition from coexisting tetragonal (T) and monoclinic (M) phases at *E*_1_ to a predominant M structural phase at *E*_2_. For bulk BTO crystal at room temperature without an external electric field, the stable domain structure is T phase. In bulk, the structure of the domain wall is usually neglected because of its very small thickness (about 5–20 nm). In such cases, the reported thickness of 90° domain wall is ~14 nm. However, in our nanoparticle with size of 160 nm, our simulations indicate that the domain wall is essentially a cross-over region with width of ~10–20 nm and is monoclinic in structure. The signature of this monoclinic cross-over region in reciprocal space is given by the splitting of the Bragg peaks as shown in our measured coherent X-ray diffraction patterns (Fig. 1 and Supplementary Fig. 3). This splitting becomes centrosymmetric at electric field *E*_2_ (223 kV cm^−1^) since the entire particle becomes predominantly monoclinic (Supplementary Note 3 and Supplementary Fig. 11). The simulated 2D polar distribution in the central slice is plotted in Fig. 3d with color representing **P**_111_. Figure 3d for field *E*_1_ = 0 kV cm^−1^ shows the coexistence of T and M phases with a vortex core at the center. For *E*_2_ = 223 kV cm^−1^, the field induced T to M phase transition leads to an M phase with the vortex core displaced to the edge of the particle. Finally, when the field is decreased to zero (*E*_3_ = 0 kV cm^−1^), the core returns to the center of the particle but the polarization domain morphology is not the same as that for *E*_1_ = 0 kV cm^−1^. This difference reflects the non-linearity of the polarization response. We observe an average polarization of 0.18 C m^−2^ at *E*_3_, comparable to bulk spontaneous values^38^ of 0.23 C m^−2^. This allows us to postulate that the non-linear behavior of the polarization response is truly governed by the local vortex structure and behavior.

### Volumetric morphology and evolution of the vortex core

**Fig. 4:**
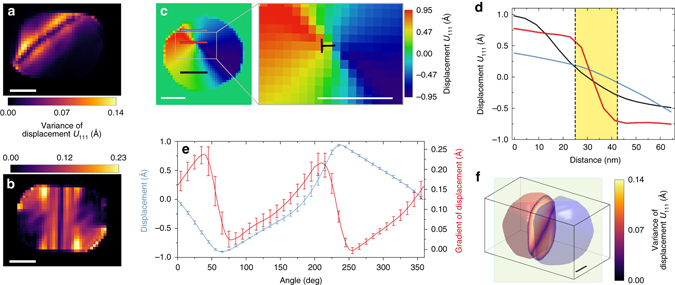
Identification of the domain wall and 3D rendering of nanorod as a defect within the nanoparticle. **a** Variance of displacement in the nanoparticle under the maximum electric field *E*_2_ (223 kV cm^−1^), calculated in the vicinity of the domain wall. **b** Variance of displacement in the nanoparticle under the electric field *E*_3_ (0 kV cm^−1^) calculated in the vicinity of the domain wall. **c** Map of displacement values and magnification of the boxed region for the nanoparticle at the slice shown as *green plane* in **f** for the state at field *E*_2_ (223 kV cm^−1^). **d** Displacement values as a function of position of lineplots in **c**. **e** Angular dependence of the displacement field and of the gradient of the displacement measured along the defect line in **f** with *error bars* indicating standard deviation over the slices in the particle. **f** Rendering of the particle under field *E*_2_ shows the 3D nanorod as a defect line whose 2D cross-section corresponds to the vortex core. *Scale bars* correspond to 60 nm

The sensitivity of BCDI to whole volume (Supplementary Fig. 7) allows us to not only track the evolution of competing polarization states under an electric field but also to identify boundaries (domain walls) separating these states and their role in polarization and vortex evolution (Fig. 3d). The vortex core forms a nanorod in the 3D nanoparticle (Fig. 4a, f). Since the diameter of this nanorod falls within the limit of our spatial resolution of 20 nm (as given by the phase retrieval transfer function in Supplementary Fig. 5), we used the variance of the displacement to study the 3D morphology of the vortex core as a 30 nm thick intrinsic paraelectric nanorod (Fig. 4a, b). The variance analysis also allows us to confirm that the vortex core coincides with regions of intersecting domain walls (Supplementary Fig. 8). The mobility of the domain walls under an applied electric field translates to a transformation of the vortex core. To estimate the number of domain walls that intersect to form the vortex core, we count the number of zeros in the angular dependence of the displacement field around the vortex (Fig. 4e). The two zeros of the displacement in Fig. 4e (*blue curve*) indicate the presence of one nanorod and hence only one vortex core within the FE nanoparticle. In the absence of an external electric field, the nanorod is in the center of the particle as shown in Fig. 4b. When the field is increased to 223 kV cm^−1^ (*E*_2_), the nanorod rotates (Fig. 4a) in the plane as the vortex core moves to the edge of the particle as confirmed by our phase-field simulation results in Figs 2d and 3d. As predicted by our simulations in Fig. 3e, if we continue to increase the field beyond 270 kV cm^−1^, the vortex core will finally disappear and the nanorod can be thought of as being erased.

**Fig. 5:**
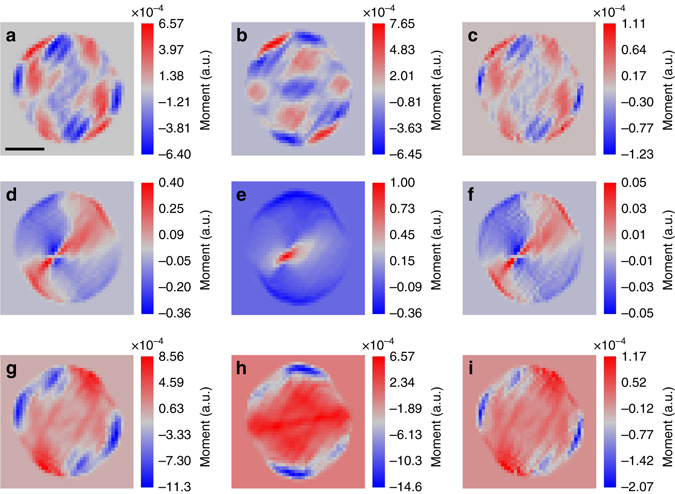
Electrically controllable chirality of the toroidal moment. Projections of the toroidal moment of the ferroelectric displacement (polarization), *T*_*x*_(**r**), *T*_*y*_(**r**), and *T*_*z*_(**r**) when the particle is subjected to a cyclic external electric field: *E*_1_ (0 kV cm^−1^), *E*_2_ (223 kV cm^−1^) and back to remnant *E*_3_ (0 kV cm^−1^). **a** Shows the projection *T*_*x*_(**r**), **b** *T*_*y*_(**r**), and **c** *T*_*z*_(**r**) under field *E*_1_ (0 kV cm^−1^). At the maximum field of *E*_2_ (223 kV cm^−1^) the projections *T*_*x*_(**r**), *T*_*y*_(**r**), and *T*_*z*_(**r**) are shown in **d**, **e** and **f** respectively. When the field is returned to *E*_3_ (0 kV cm^−1^) the projections *T*_*x*_(**r**), *T*_*y*_(**r**), and *T*_*z*_(**r**) are shown in **g**, **h** and **i** respectively. Each projection of the moment can be seen as a new ferroelectric phase within a single FE particle with electrically controllable chirality. The displayed view of the particle is in the plane perpendicular to the vortex core. For more views please see the Supplementary Movie 1. *Scale bars* correspond to 60 nm

Since the summation of Born effective charges allows us to scale the displacements with the local polarization within the particle, Eq. 1 allows us to use the zeros of the displacement gradients (*red curve* in Fig. 4e) to estimate the nature and number of domain walls and hence FE multiple states for any given slide and component of the polarization within our resolution limit. The regions where the displacement gradient changes sign indicates transition across the domain wall (Fig. 4c–e). Using this analysis, the orientation and behavior of the polarization vector across the domain wall (Supplementary Fig. 9), and from the locations of the first and second zeros in Fig. 4e, we determine a 173 ± 10° domain wall parallel to the spontaneous polarization of the adjacent domains. This is in good agreement with the predicted value of 180° for Bloch walls (Supplementary Note 4 for more information). To compare with our experimental tracking of the vortex structure under an external electric field, our phase-field model in Fig. 3e predicts that at fields above 270 kV cm^−1^, the toroidal moment disappears leaving the axial polarization as the only non-zero order parameter. This disappearance indicates a vortex-to-polarization transformation^10, 37^ in the nanoparticle making it useful for NRAM applications. However, for other potentially new applications such as tunable optical behavior, electrically controllable chirality of the toroidal moment is essential (Fig. 5).

### Chirality of the three-dimensional toroidal moment

In Fig. 5, we used the distribution of the toroidal moment of the polarization given by ${\bf{T}}(r) = \nabla \times {{\bf{u}}_{111}}(r)$ as the order parameter of the electric-field induced phase transition^10^. Since BCDI provides 3D volume information, we can conveniently retrieve the spatial distribution (Fig. 5) of the projections of the toroidal moment, *T*_*x*_(**r**), *T*_*y*_(**r**), and *T*_*z*_(**r**). The magnitude gives the amount of circulation at a given point while the directions (Fig. 5) allow us to understand the chirality of the vortex structure under electric field. Electrically controllable chirality of the toroidal moment will be achievable if the projections along the *x*, *y*, and *z* directions given by *T*_*x*_(**r**), *T*_*y*_(**r**), and *T*_*z*_(**r**) are non-superposable on each other under the action of an external field (Supplementary Movie 1). Figure 5 shows that at electric field *E*_2_ = 223 kV cm^−1^, *T*_*x*_(**r**) and *T*_*y*_(**r**) are superposable and are mirror images of each other while *T*_*z*_(**r**) breaks the symmetry and is non-superposable to the other projections. Also, *T*_*x*_(**r**), *T*_*y*_(**r**) and *T*_*z*_(**r**) are at least four orders of magnitude larger than those at states *E*_1_ *=* *E*_3_ *= *0.0 kV cm^−1^. At field *E*_1_ *= *0 kV cm^−1^ the directions of toroidal moment *T*_*x*_(**r**), *T*_*y*_(**r**), and *T*_*z*_(**r**) can easily be approximated as mirror images of one another with noticeable difference in the magnitude. As the field increases to *E*_2_, the symmetry is broken in the plane of the applied electric field. When the field is reduced to *E*_3_, the symmetry is restored and a new toroidal phase is obtained for *T*_*x*_(**r**), *T*_*y*_(**r**), and *T*_*z*_(**r**). Unlike in earlier studies of FE that used the spontaneous polarization as evidence of phase transition, we observe that the chirality of the toroidal moment can also be used as an order parameter^10^ due to the possibility of controlling it using electric fields (Fig. 5). This may open exciting opportunities for memory devices, nanomotors, nanotransducers, nanoswitchers, nanosensors, and so on.

## Discussion

In summary, using BCDI we have studied in operando the behavior of a 3D vortex in a 160 nm ferroelectric nanoparticle formed as a result of competing interactions involving ferroelectric domains. We have captured a 30 nm thick intrinsic nanorod whose 2D cross-section serves as the vortex core. Although the nanoparticle is ferroelectric, the nanorod has a paraelectric phase which﻿ extends across the particle volume. This rod is also mobile under an applied electric field. The vortex core is displaced from the particle’s center to its edge and returns to particle’s center when the field is switched off. Also, the toroidal moment has an electrically controllable chiral behavior which can potentially be used to produce a tunable optical behavior^27^. Our simulations also predict that the toroidal moment is accompanied by a structural transformation under the influence of the field from a state of coexisting tetragonal and monoclinic polarization domains to one that is purely monoclinic. This has not been seen in electronic structure based calculations as the scale of our nanoparticle is much larger. Under zero electric field (Figs 2c and 3a, for examples), we identify two distinct transitions as the diameter of the nanoparticle increases: from a quadruplet polar domain to a multiplet state. As the electric field across the particle is increased to 223 kV cm^−1^ (Figs 2c and 3b, for examples), we observe two domains within the entire nanoparticle. It is also evident that the walls separating these domains intersect to form the vortex core with an electrically controllable chirality of the toroidal moment (Fig. 5 and Supplementary Movie 1).

Our observed complex topologies of polarizations can be engineered and controlled by applied electric fields, stresses, as well as by built-in interface effects at the mesoscale. Since the vortex core can be displaced and erased through a reversible hysteretic transformation path, it can be thought of as a conductive channel inside a monolith of nominally insulating ferroelectric material. This could be exploited in the design of integrated electronic devices based on polar vortices and the possibility of creating artificial states of matter through the control of related phase transitions. Advances in the development of bright coherent light sources allow greater temporal resolution in dynamical studies of these phenomena. Our findings can help to pave the way for future studies of shape and size dependence along with hysteretic behavior under temperature and other external perturbations to unveil what is yet hidden in vortex dynamics.

## Methods

### Powder preparation

BaTiO_3_ (BTO) nanoparticles were synthesized in the sol-gel-hydrothermal reaction of BaCl_2_ and a TiCl_4_ solution in oxygen atmosphere. The typical procedure consisted of diluting 1.1 ml of TiCl_4_ in 2.3 ml of HCl_2_ to form a yellowish solution. This solution was later mixed with 2.4 g of BaCl_2_ 2H_2_O and dissolved in 15 ml of deionized water to form a solution of barium titanium. While stirring until bubbles of N_2_ is emitted, 13 ml of NaOH 6 mol was added to the barium titanium solution, to produce uniform colloidal barium titanium slurry. This colloidal solution was transferred into a 500 ml Teon-lined stainless autoclave and heated at temperature ranges of 70–220 °C for 3 h. The entire growth procedure was developed in air with sealed autoclave which was later evacuated and filled with pure oxygen. The pressure was controlled during the experiment at a fixed value of 60 bar. At the end of the reaction, the autoclave was naturally cooled to room temperature with the formation of solid white powder BTO attached to the bottom and inner wall of the Teon container. This powder was later centrifuged and washed with distilled water and ethanol to remove remaining ions, and dried at 60 °C for 6 h in vacuum.

### External electric field control

We used ferroelectric testing system to perform programmable application of the electric field as well as in-situ feedback monitoring of the sample. The system is composed of such elements as low noise and high sensitivity electro-meter amplifier, precision potentiometer, circuit integrated reference capacitors, and integrating capacitors, as well as leakage impedance compensators. The system can perform ferroelectric tests in compliance with the IEEE standard 180 as well as simultaneously driving the sample with stable pulses through integrated function generator. The system has 18-bit ADC capable of 76 µV sensitivity at capture rate of 0.5 µs. The maximum frequency of applied pumping pulses with continuous hysteresis measurements feedback is 250 kHz. The testing system is fully programmable allowing algorithmic on-line control of the experimental parameters based on the readings.

### Ferroelectric capacitor

The scheme of the ferroelectric capacitor used in these measurements is shown in Supplementary Fig. 1. The BTO powder was first pre-characterized with laboratory X-ray diffraction (XRD) (Supplementary Fig. 2), optical and scanning electron microscopy (Supplementary Fig. 10). The sample shows expected tP5 crystal structure. The uniformity of particles as seen in Supplementary Fig. 10 ensures that when a single peak is detected during the measurements in (111) Bragg reflection, this peak corresponds to a single nanoparticle.

The BTO nanoparticles were thoroughly mixed with conductive polymer matrix. The volume fraction of incorporated BTO nanoparticles was 15%. The polymer matrix was prepared in advance by mixing liquid bisphenol A based epoxy with conducting carbon nanoparticles (40% by volume). The composite solution was transferred into template with dimensions 2 × 2 × 1 (mm × mm × mm) and cured with commercially available agent in an oven first at 90 °C for 1 h and then at 60 °C for 4 h. Gold electrodes were sputtered on corners of two sides of the sample and thin conductive Kapton film was attached with insulating surfaces facing in the outward direction. Dispersed particles are known to form conducting chains in epoxy polymers^39, 40^. This was used to deliver the current directly to embedded BTO nanoparticles. During the experiments voltage scans of 30 cycles (Supplementary Figs 3 and 4) were performed with monitoring of evolution of diffraction pattern to identify active (linked in the conductive chain) and inactive (insulated in the epoxy matrix) BTO nanoparticles. When the diffraction pattern showed no response to the applied voltage over the scan it is assumed to be isolated from the conducting particles. Inactive BTO nanoparticles can be potentially used for ex situ studies and alignment procedures during the experiment.

### Experimental procedure

A Si (111) monochromator was used to tune into X-rays with energy of 9 keV with 1 eV bandwidth monochromaticity and 0.7 µm transverse coherence length. The selected X-rays were then focused downstream from the monochromator by a pair of Kirkpatrick-Baez mirrors to achieve beam size of 700 nm by 700 nm. We used motorized arm of Kappa diffractometer to position Medipix2 CMOS X-ray detector around the diffraction sphere to record diffraction pattern at characteristic (111) Bragg reflection of BTO. The Medipix2 detector has pixel size of 55 µm by 55 µm with the sensor composed of 256 by 256 picture elements all working in a single photon counting mode. The detector was positioned at 0.8 m distance from the sample to ensure enough resolution of the interference pattern. To decrease the loss of the scattered photons an evacuated flight tube was placed between the sample and the detector. The high sensor gain of the detector and the use of flight tube allowed effective capture the photons scattered by the sample structures and the single counting mode of the detector allowed better discrimination of the signal from background noise, which all is crucial for such photon starving techniques as nanoscale BCDI. The rocking curve was collected as series of 2D diffraction patterns in the vicinity of the Bragg peak at 2*θ* = 34.55° with the scanning range of ∆*θ* = ± 0.6°. Throughout a single rocking curve scan a total of 140 patterns were collected. The dataset for the virgin state *E*_1_ was collected before cycling the capacitor. The following maximum state *E*_2_ was measured after 30 cycles of applied and released electric field and the final zero field *E*_3_ was recorded right after the saturation field was released.

### Reconstruction procedure

In order to obtain 3D real-space images of the electric-field induced structural and polarization changes within a single nanoparticle, it is necessary to invert the coherent diffraction pattern using a 3D Fourier transform. However, this requires knowledge of not only the full set of measured diffraction amplitudes, but also the phases of the complex wave field for the diffraction data. Iterative phase retrieval algorithms based on Fienup’s Hybrid Input–Output (HIO) method^41^ were utilized. Inverting the diffraction data is a critical step that uses a computer algorithm that takes advantage of internal redundancies when the measurement points are spaced close enough together to meet the oversampling^42^ requirement. The first step is to postulate a 3D support volume in which all the sample density will be constrained to exist. These methods make use of iterative Fourier transforms between the data and its real-space image, applying both support and phase-range constraints to the latter^43^.

The coherent X-ray diffraction data in Fig 1, Supplementary Figs. 11 and 3 have 12 pixels per fringe in the horizontal and 18 pixels per fringe in the vertical directions. This implies that data can be binned 5 (7) times and still satisfy oversampling with respect to a boxed shaped support of 33 × 48 × 30 pixels inside of an array of Fast Fourier Transform of 64 × 96 × 128 pixels. The oversampling of the data used for phasing was therefore 2.2 in the horizontal and 2.6 in the vertical^44^ although this found to have little effect on the results.

A total of 3800 iterations of HIO were performed on the coherent diffraction data in the virgin state (*E*_1_) to obtain the 3D (2D slices) images of the nanoparticle in Fig. 2d with a real-space phase constrained in the range [−*π*; *π*]. This phase was unwrapped to obtain the displacement field which was projected on single isosurface contoured at 35% of the maximum density. For the non-zero electric field cases an average of 4100 iterations of HIO were performed.

It is evident from the asymmetries in our measured diffraction patterns in Supplementary Fig. 3 that the difference Fourier approximation is appropriate, so long as the data are correctly centered^45^. To confirm the reproducibility and uniqueness of the obtained solutions, we performed a series of phase retrieval procedures with different random input phases for the measured scattered radiation. We defined a cost function in the form of an error metric as

2 $$E_k^2{\rm{ = }}\frac{{\mathop {\sum}\nolimits_{i = 1}^N {{{\left( {\left| {F_i^{{\rm{sim}}}} \right| - \sqrt {I_i^{\exp }} } \right)}^{\!\!2}}} }}{{\mathop {\sum}\nolimits_{i = 1}^N {I_i^{\exp }} }},$$

where $\left| {F_i^{{\rm{sim}}}} \right|$ is the magnitude of the simulated amplitude and $I_i^{\exp }$ is the experimental intensity of point *i* in the reciprocal space map. More information on the reconstruction can be found in Supplementary Note 1.

The phasing process, for a given initialization of the algorithm, is determined to be complete when the error metric is reduced to 10^−12^. The maximum number of iterations allowed was typically about 4100. The differences between many equivalent solutions obtained from random initial phases provide a measure of the resolution of the resulting reconstruction in the form of a phase retrieval transfer function (PRTF)^46, 47^. The final resolution of 18 nm was obtained from computing the PRTF (Supplementary Fig. 5).

### Phase-field model

To model the particle behavior and confirm the evolution of the vortex manifested through change in polarization we used phase-field simulations. A 48 × 48 × 48 grid points mesh with periodic boundary conditions has been used for the simulation of a nanoparticle embedded in non-polarizable medium, since the shape of the nanoparticle can affect the domain pattern, in the core part of 15 × 24 × 15 grid points we make a BTO nanoparticle with a profile which is close to the experiment, as shown in Supplementary Fig. 6 and the outer part is the non-polarizable medium, the Landau parameters for BTO taken from refs ^38, 48^, the elastic coefficients are shown in the Supplementary Table 1 and the relative background permittivity *ε*_*b*_ = 100. Phase-field simulations also show that the formation of a vortex in BTO nanoparticle is governed by the competing energies and an applied electric field can modulate the established balance and alters the energy landscape (Supplementary Fig. 12). More information on phase-field modeling can be found in Supplementary Note 2.

### Data availability

Raw data were measured at the Advanced Photon Source Sector 34-ID-C and are permanently deposited there. The data supporting the findings of this study are available from the corresponding author upon request.

## Electronic supplementary material

Supplementary Information

Supplementary Movie 1
