# Supplementary material for: Three-dimensional imaging of vortex structure in a ferroelectric nanoparticle driven by an electric field
Source: Nat Commun. 2017 Aug 17;8:280. doi: 10.1038/s41467-017-00318-9 (PMC5561144; doi:10.1038/s41467-017-00318-9)
Supplement: Supplementary file 1 — Supplementary Information [file 41467_2017_318_MOESM1_ESM.pdf]

File Name: Supplementary Information
Description: Supplementary Figures, Supplementary Table, Supplementary Notes and Supplementary References.

File Name: Supplementary Movie 1
Description: Projections of particle's toroidal moment at different views for different applied electric fields.

Supplementary Information

Supplementary Note 1: Difference map algorithm

The difference map technique is effectively used in protein crystallography experiments for the identification of localized small changes in active sites of molecules[1]. This approach is based on the assumption that the reciprocal-space phases of the diffraction pattern changes uniformly after relatively small structural transitions, which can be identified through correlations of intensity redistribution along the fringes. It was shown that the difference Fourier map is able to detect much smaller features of electron density than those revealed by a normal Fourier map with the same phases[1]. This approach can therefore be sensitive to smaller changes within the crystal electronic density that are related to second order displacement gradient effects such as polarization induced by structural phase transformation under the influence of an electric field. A relative change in the diffraction intensity in the vicinity of a reciprocal lattice point caused by the application of the electric field from E_0 to E, at a given X-ray photon energy can be expressed as $\Delta I(E,\mathbf{q}) = \left[I(E,\mathbf{q}) - I(E_0,\mathbf{q}) \right] / I(\mathbf{q})$, with the intensity distribution for the nanoparticle in the virgin state given by:

$$I(\mathbf{q}) \sim \left| \int d^3\mathbf{r} \rho_{\mathbf{G}_{111}}(\mathbf{r}) \exp\left(-i\mathbf{G}_{111} \cdot \mathbf{u}_{111}\right) \exp\left(-i\mathbf{q} \cdot \mathbf{r}\right) \right|^2 \quad (1)$$

The resulting difference Fourier density in real space, $\Delta\rho(\mathbf{r})$, is the difference of the two complex density functions, given by the Fourier transform of the differences between the coherent diffraction patterns, using the phased diffraction of one of them as the common phase function $\psi(\mathbf{q})$:

$$\Delta\rho(\mathbf{r}) = \int \left[\sqrt{I(E,\mathbf{q})} - \sqrt{I(\mathbf{q})} \right] e^{-i\psi(\mathbf{q})} e^{i\mathbf{q}\cdot\mathbf{r}} d^3\mathbf{q} \,. \quad (2)$$

The deviation of the momentum transfer $\mathbf{Q}$, from the reciprocal lattice vector, $\mathbf{G}_{111}$ is given by $\mathbf{q} = \mathbf{Q} - \mathbf{G}_{111}$ for the measured (111) Bragg peak. The ionic displacement map can be obtained from the resulting complex function, $\Delta\rho(\mathbf{r})$. Changes in crystal strain, when the shape and density are constant, should appear mainly in the imaginary part of $\Delta\rho(\mathbf{r})$.

Supplementary Note 2: Phase-field simulation

Recent theoretical studies based on phase field simulations have predicted the presence of vortices in BTO[2] and other ferroelectric nanostructures[3,4] and the possibility to control such vortices using both homogeneous and inhomogeneous electric fields[5,6]. In the phase field simulation, the domain structure of the nanoparticle can be described by the spatial distribution of polarization $\mathbf{P}(P_1, P_2, P_3)$, the total free energy of the system F can be written as: $F = \int dV\left(f_{\mathrm{L}} + f_{\mathrm{ela}} + f_{\mathrm{g}} + f_{\mathrm{e}}\right)$, where f_{L} is the Landau-Devonshire free energy density, f_{ela} is the elastic energy density, f_{e} is the electrostatic energy density and f_{g} is the gradient energy density, respectively. For ferroelectrics with perovskite structure the Landau-Devonshire energy density f_{L} can be expressed as:

$$\begin{aligned} f_L = {} & \alpha_1 \sum_i P_i^2 + \alpha_{11} \sum_i P_i^4 + \alpha_{12} \sum_{i>j} P_i^2 P_j^2 + \alpha_{111} \sum_i P_i^6 + \alpha_{112} \sum_{i>j} \left(P_i^4 P_j^2 + P_i^2 P_j^4\right) \\ & + \alpha_{123} \prod_i P_i^2 + \alpha_{111} \sum_i P_i^8 + \alpha_{112} \sum_{i>j} \left(P_i^6 P_j^2 + P_i^2 P_j^6\right) \\ & + \alpha_{1122} \sum_{i>j} P_i^4 P_j^4 + \alpha_{1123} \sum_{i \neq j \neq k, j>k} P_i^4 P_j^2 P_k^2 \end{aligned} \quad (3)$$

where α_i, α_{ij}, α_{ijk}, and α_{ijkl} are the Landau parameters that can get from the experiments. The elastic energy density can be written as $f_{\mathrm{ela}} = \frac{1}{2} C_{ijkl} \left(\varepsilon_{ij} - \varepsilon_{ij}^0\right)\left(\varepsilon_{kl} - \varepsilon_{kl}^0\right)$, where C_{ijkl} is the elastic stiffness tensor, ε_{ij} is the total strain, and $\varepsilon^0_{ij} = Q_{ijkl} P_k P_l$ is the spontaneous strain during the phase transformation, in which Q_{ijkl} is the electrostrictive coefficient. The elastic stiffness tensor is assumed to be equal in the nanoparticle and non-ferroelectric matrix. The electrostatic energy can be expressed as $f_{\mathrm{e}} = 1/2(\mathbf{E} \cdot \mathbf{P})$, where $\mathbf{E}$ is the electrostatic field in the nanoparticle, the electric field can be evaluated from the electrostatic potential φ as $\mathbf{E} = -\nabla \varphi$. In the model the space is assumed to be charge free, therefore the equation:

$$\nabla \cdot \left(-\varepsilon_0 \varepsilon_{\mathrm{b}} \nabla \varphi + \mathbf{P}\right) = 0 \quad (4)$$

can be used to solve the electric field, in which ε_0 and ε_{b} are permittivity of vacuum and relative background permittivity, respectively. The gradient energy density can be obtained by $f_{\mathrm{g}} = \frac{1}{2} G_{ijkl} P_{i,j} P_{k,l}$, where G_{ijkl} is the gradient coefficient. And in this model, the size effect of the free energy in the nanoparticle is not considered in the simulation. In the phase field model, the temporal evolution of the polarization can be obtained by solving the time-dependent Ginzburg- Landau equation[7]:

$$\frac{\delta P_i(\mathbf{r},t)}{\delta t} = -L \frac{\delta F}{\delta P_i(\mathbf{r},t)}, \ (i = 1,2,3) \quad (5)$$

where r is the spatial vector and L is the kinetic coefficient. We have simulated the evolution of the polarization in the nanoparticle, while maintaining a fixed polarization $\mathbf{P} = \varepsilon_{\mathrm{m}} \varepsilon_0 \mathbf{E}$ for different external electric fields E in the matrix.

Supplementary Note 3: Analysis of coherent X-ray diffraction signature due to structural phases

The monoclinic phase of the nanoparticle has noticeable influence on the analysis of our Bragg coherent X-ray diffraction in reciprocal space. The signature of this monoclinic cross-over region in reciprocal space contributes to the asymmetry and splitting (modulation) of the Bragg peaks as shown in our measured coherent X-ray diffraction (CXD) patterns (see Figure 1, Supplemental Figure 3 and Supplementary Figure 11).

To understand these signatures in reciprocal space measured in the vicinity of the (111) Bragg peak, let us consider that the elastic and ferroelectric displacement field within the nanoparticle is given by $\mathbf{u} = \mathbf{u}_{111}$. Within the kinematical limits and first Born approximation[8], the scattered X-ray intensity distribution at the detector plane obtained from the nanoparticle can be given as:

$$I(\mathbf{q}) \sim \left| \int_V \rho_{\mathbf{G}_{111}}(\mathbf{r}) \mathrm{e}^{-i\mathbf{G}_{111}\cdot\mathbf{u}_{111}} \mathrm{e}^{-i\mathbf{q}\cdot\mathbf{r}} d^3\mathbf{r} \right|^2 = \left| \int_V \tilde{\rho}(\mathbf{r}) \mathrm{e}^{-i\mathbf{q}\cdot\mathbf{r}} d^3\mathbf{r} \right|^2 , \quad (6)$$

where $\rho_{\mathbf{G}_{111}}(\mathbf{r})$ is the ideal real-valued Bragg electron density distribution within the nanoparticle and $\tilde{\rho}(\mathbf{r}) = \rho_{\mathbf{G}_{111}}(\mathbf{r}) \mathrm{e}^{-i\mathbf{G}_{111}\cdot\mathbf{u}_{111}}$ represents the complex-valued strained state of the ferroelectric BTO nanoparticles. For brevity, the intensity distribution equation can be interpreted as the magnitude squared of the Fourier transform from a complex-valued density function:

$$I(\mathbf{q}) = \left| \mathrm{FFT}[\tilde{\rho}(\mathbf{r})] \right|^2 . \quad (7)$$

Under electric field E_2 (223 kV cm^{-1}), the nanoparticle is in the monoclinic phase with displacement field given by $\mathbf{u}_{111} = \mathbf{u}_{\mathrm{M}}$. This implies that the splitting of the Bragg peak (see Supplementary Figure 11) under this field is strongly modulated by the magnitude squared of the Fourier transform of the complex density function:

$$\tilde{\rho}_{\mathrm{mono}}(\mathbf{r}) = \rho_{\mathbf{G}_{111}}(\mathbf{r}) \mathrm{e}^{-i\mathbf{G}_{111}\cdot\mathbf{u}_{\mathrm{M}}} . \quad (8)$$

This diffraction pattern is centrosymmetric about the center 0th order peak, with the inverse of 1st and -1st order satellite peaks separation related to the ordering of this monoclinic phase. Under field E_1 (0 kV cm^{-1}) and E_3 (0 kV cm^{-1}) the particle undergoes a structural phase transition exhibiting the coexisting tetragonal (T) and monoclinic (M) phases. Under these two electric field cases, the displacement field within the nanoparticle can be expressed as $\mathbf{u}_{111} = \mathbf{u}_{\mathrm{M}} + \mathbf{u}_{\mathrm{T}}$. This implies that the nanoparticle complex electron density becomes $\tilde{\rho}(\mathbf{r}) = \rho_{\mathbf{G}_{111}}(\mathbf{r}) \mathrm{e}^{-i\mathbf{G}_{111}\cdot(\mathbf{u}_{\mathrm{T}} + \mathbf{u}_{\mathrm{M}})}$. This cross-over between the M and T phases provides a different type of modulation and asymmetry to the Bragg peak (see diffraction patterns in Supplementary Figure 11) than the one observed in the purely monoclinic phase. In our analysis, we considered the diffraction patterns at E_1 and E_3 as a perturbation to the pure monoclinic phase at E_2 given by:

$$\tilde{\rho}(\mathbf{r}) = \rho_{\mathbf{G}_{111}}(\mathbf{r}) \mathrm{e}^{-i\mathbf{G}_{111}\cdot(\mathbf{u}_{\mathrm{T}} + \mathbf{u}_{\mathrm{M}})} = \rho_{\mathbf{G}_{111}}(\mathbf{r}) \mathrm{e}^{-i\mathbf{G}_{111}\cdot\mathbf{u}_{\mathrm{M}}} \mathrm{e}^{-i\mathbf{G}_{111}\cdot\mathbf{u}_{\mathrm{T}}} = \tilde{\rho}_{\mathrm{mono}}(\mathbf{r}) \mathrm{e}^{-i\mathbf{G}_{111}\cdot\mathbf{u}_{\mathrm{T}}} . \quad (9)$$

This implies that the centrosymmetric nature of the measured Bragg peak when the particle is in the monoclinic phase becomes modulated by contributions from domain patterns of the T phase.

The subtle discrepancies in phase field simulation and experimental reconstructions (see Figure 2) can be attributed to the experimental noise, residual strain, and other physical features within the nanoparticle not accounted for by the phase field model.

Supplementary Note 4: Analysis of structural phase transformation

By applying a cyclic electric field to the capacitor (Supplementary Figure 3), we induce a reproducible phase transformation. This is evident by the splitting of the (111) reflection along different Debye-Shearer rings corresponding to d-spacing of 2.43 Å, 2.32 Å, and 2.22 Å. To understand the nature of these structural phases and the mechanisms controlling their nucleation, we perform 3D phase-field simulations while monitoring the evolution of the minimum free energy density of polarization states within the nanoparticle (Supplementary Figure 11). We focus our attention on the ferroelectric polarization state and its distribution within the particle at fields E_1 (Fig. 3A), E_2 (Fig. 3B) and E_3 (Fig. 3C). At electric field E_1 the state of the particle shows phase co-existence of tetragonal (T) and monoclinic (M) structures. As we increase the external electric field to an experimentally allowed maximum of 10V, the T phase nucleates into a predominant M-phase (Fig. 3D). Decreasing the field to the remnant state transforms the particle back to a mixture of T+M phases with noticeable hysteresis in polarization. Phase field modeling predicts that if the electric field is large enough (the saturation field), the polarization will be poled to adopt the R phase.

We distinguish between the various forms of domain walls by comparing them with idealized profiles of boundaries separating nanodomain states with opposite polarization, *i.e.* 180 degree domain walls. The idealized polarization profiles in Supplementary Figure 9 depict that Bloch domain walls are chiral and polarization reversal is achieved by polarization rotation within the plane of the domain wall while in contrast, for ideal Ising wall the polarization remains strictly parallel or antiparallel to the spontaneous polarization of the adjacent domains.

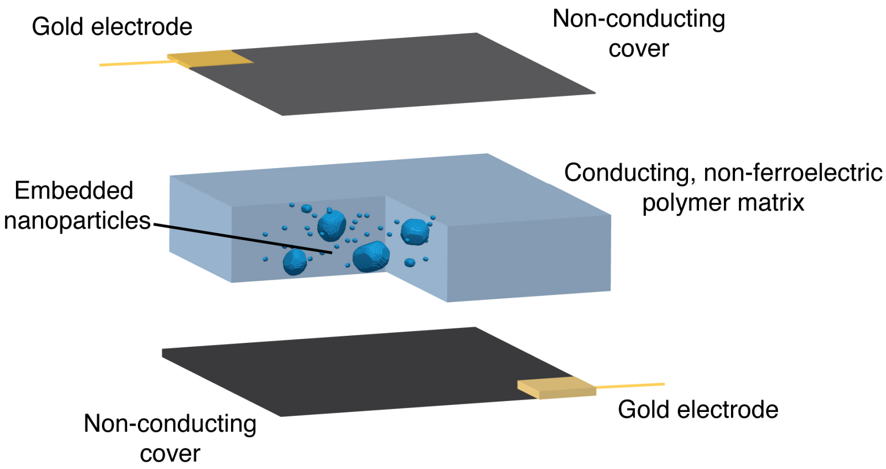

Supplementary Figure 1: Scheme of the capacitor. The BTO nanoparticles have been placed in a non-polarizing medium. Gold electrodes were later sputtered on the matrix providing outlets for wire bonding. The high penetrating abilities of X-rays make the system to be virtually transparent to perform Bragg diffraction.

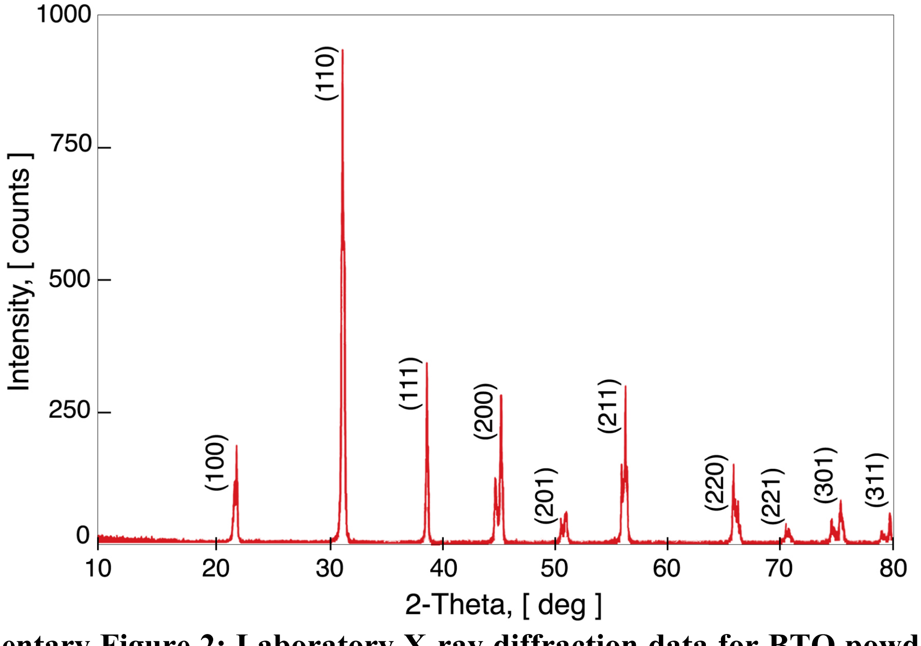

Supplementary Figure 2: Laboratory X-ray diffraction data for BTO powder. XRD diffraction shows ensemble average information on crystalline phases of the BTO nano powder. Since this data is collected using a wide X-ray beam, we can only conclude statistically on the collective crystallographic facets orientation of the nanoparticles. Bragg coherent diffraction experiment performed in this work allows us to localize a single nanoparticle.

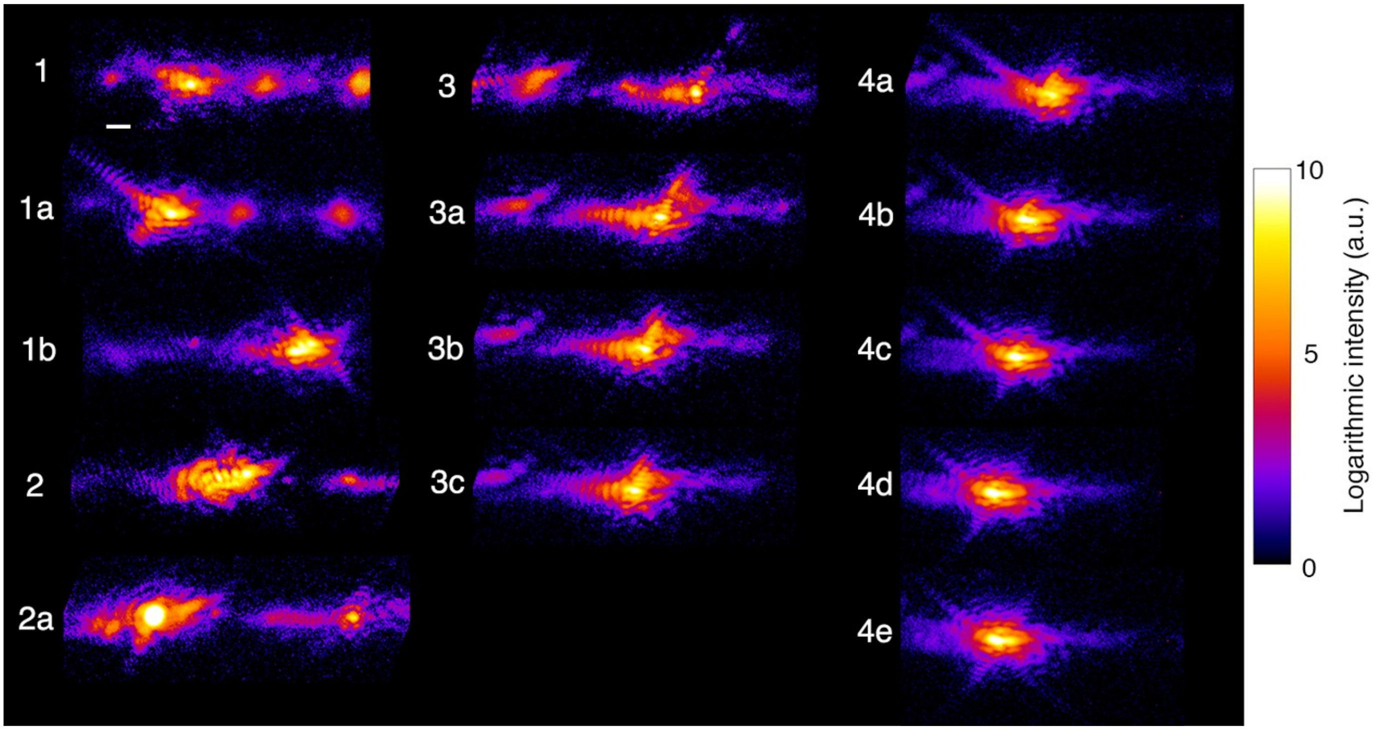

Supplementary Figure 3: Evolution of the Bragg coherent X-ray diffraction pattern under applied electric field for a single BTO nanoparticle undergoing structural phase transition. The numbering of each diffraction pattern from (1) to (4e) corresponds to the states of the nanoparticle under different values of a cyclic external electric field. Diffraction pattern at state (1) is collected under 0V, (1a) under applied field of 2V, (1b) under applied field of 8V, (2) under applied field of 9V, (2a) under applied field of 5V, (3) under applied field of 0V, (3a) under applied field of -1V, (3b) under applied field of -7V, (3c) under applied field of -10V, (4a) under applied field of -8V, (4b) under applied field of -6V, (4c) under applied field of -4V, (4d) under applied field of -2V, and (4e) under applied field of 0V. As given by phase field simulation, 10V corresponds to an electric field of 223 kV cm^{-1} across the single BTO nanoparticle. Scale bar corresponds to 0.1 $Å^{-1}$.

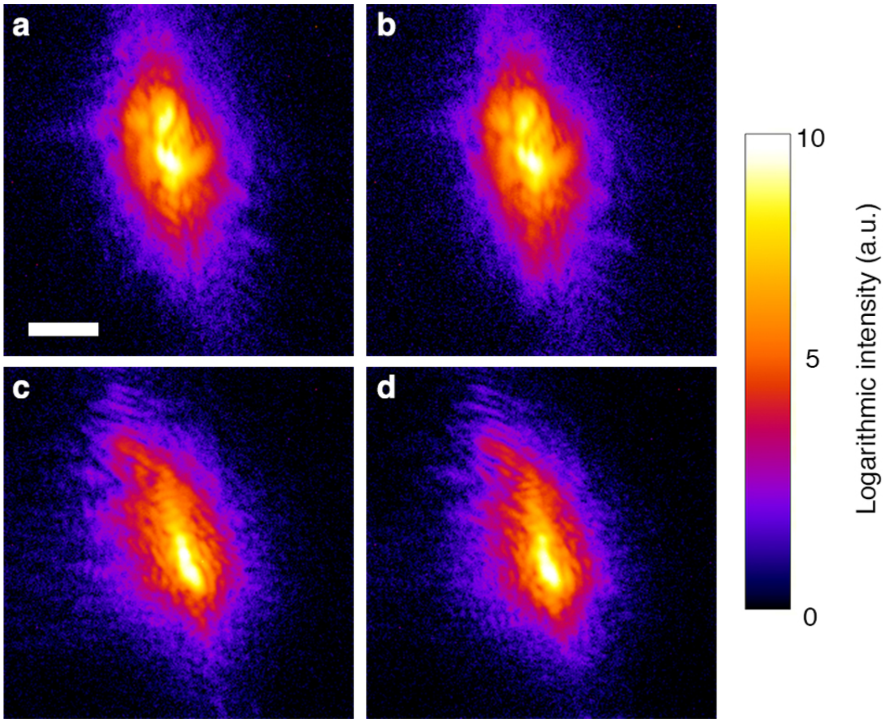

Supplementary Figure 4: Evolution of the diffraction pattern under applied electric field for two inactive BTO nanoparticles. a, particle 1 at zero applied electric field before cycling. **b**, particle 1 under maximum electric field after 30 continuous cycles. **c,** particle 2 at zero applied electric field before cycling. **d**, particle 2 under maximum electric field after 30 continuous cycles. These particles do not undergo the expected structural phase transition. Scale bar corresponds to 0.1 $Å^{-1}$.

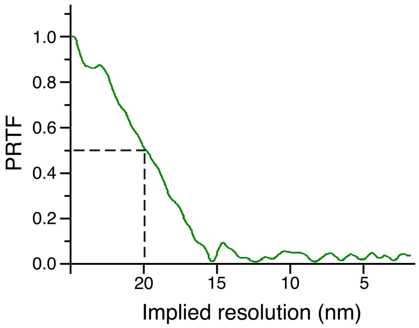

Supplementary Figure 5: Resolution estimation by phase retrieval transfer function. The phase retrieval transfer function (PRTF) is a tool that provides an accurate resolution measure. The experimental diffractions are averaged over constant reciprocal space frequency contours to produce the PRTF, which takes a value of 1 where the iterative algorithm produced perfect convergence consistently, and a value near 0 where the algorithm continually failed to converge. The dotted line at the PRTF cutoff of 0.5 (50%) is used to determine the resolution at approximately 20nm.

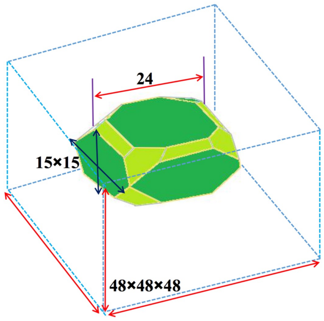

Supplementary Figure 6: Schematic diagram of nanoparticle in phase field simulation. The diagram shows the design of the particle model used in the phase field simulation. The unit is composed of 48x48x48 grid points of which the central 15x24x15 grid points is the particle of BTO itself, surrounded by the non-ferroelectric medium.

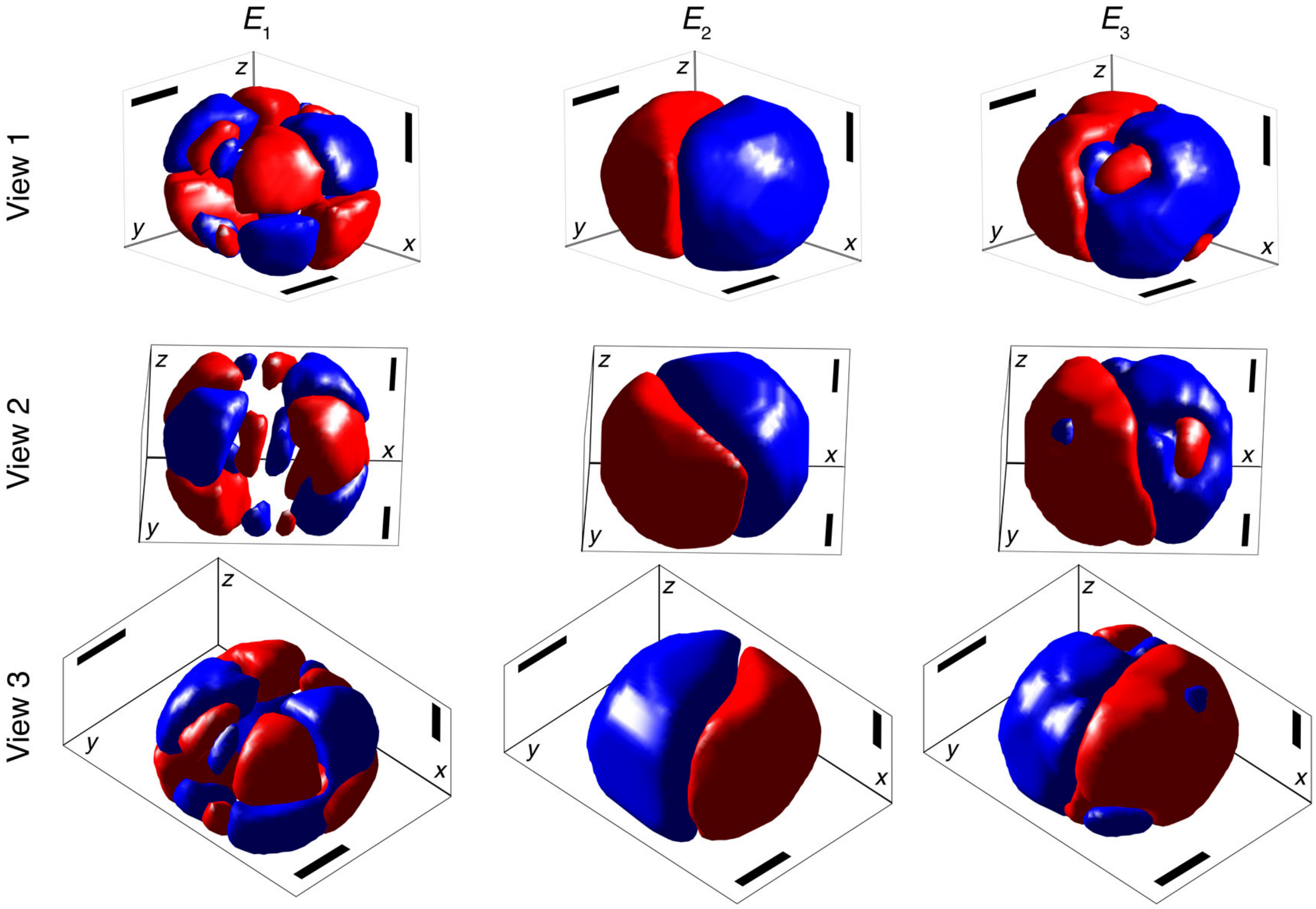

Supplementary Figure 7: Three-dimensional renderings of polarization dynamics. Isosurfaces of polarization $\mathbf{P}_{111}$ for three different applied electric fields E_1 (0 kV cm^{-1}), E_2 (223 kV cm^{-1}), and E_3 (0 kV cm^{-1}). Different 3D views are shown to highlight the morphology and evolution of poly-domain states within a single BTO nanoparticle. For fields E_1 and E_3 the blue and red isosurface color represent contour levels of the polarization drawn at -0.18 Cm^{-2} and +0.18 Cm^{-2} respectively. For field E_2, the blue and red isosurface color represent contour levels of the polarization drawn at -0.23 Cm^{-2} and +0.23 Cm^{-2} respectively. Scale bars correspond to 60 nm.

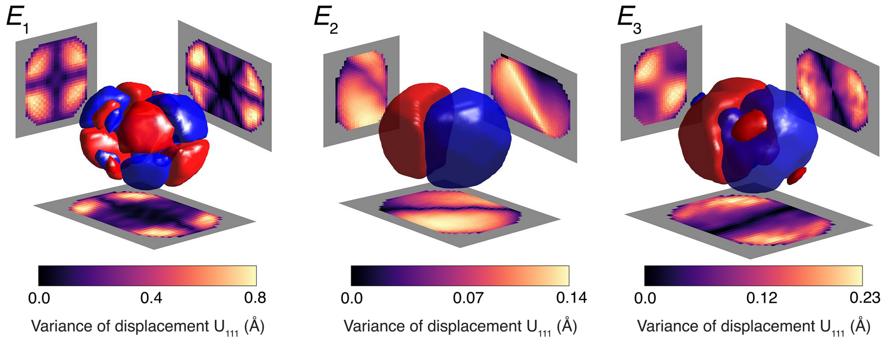

Supplementary Figure 8: Variance of the displacement field under applied electric field. Projections of variance (placed along with the 3D of polarization) for 3 applied fields E_1 (0 kV cm^{-1}), E_2 (223 kV cm^{-1}), and E_3 (0 kV cm^{-1}). Variance of the displacement fields help to assess the regions of domain wall intersection and its dynamics. For fields E_1 and E_3 the blue and red isosurface color represent contour levels of the polarization drawn at -0.18 Cm^{-2} and +0.18 Cm^{-2} respectively. For field E_2, the blue and red isosurface color represent contour levels of the polarization drawn at -0.23 Cm^{-2} and +0.23 Cm^{-2} respectively.

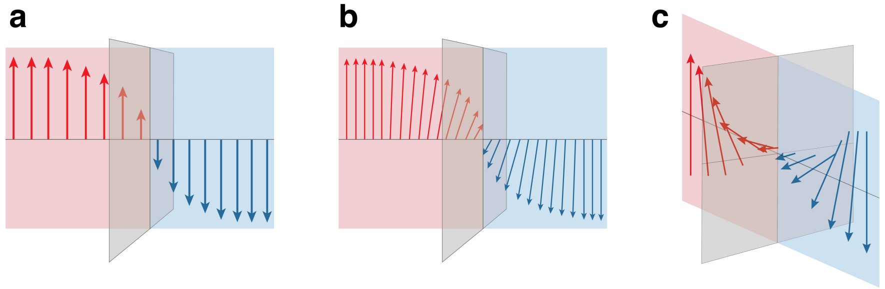

Supplementary Figure 9: Schematic of domain walls. Idealized Profiles of (**a**) Ising, (**b**) Neel and (**c**) Bloch domain walls in ferroelectrics. A ferroelectric material possesses a built-in electrical polarization that is switchable with an electric field. In contrast to ferromagnetic materials in ferroelectrics, the spontaneous electrostriction, the coupling between ferroelectric polarization and lattice strain, imposes a significant energy cost for rotating the polarization away from the symmetry-allowed directions in the crystal lattice. Thus, ferroelectric domain wall can be either Ising type, Neel and Bloch domain. The light red and blue planes are used to indicate regions of opposite spontaneous polarization.

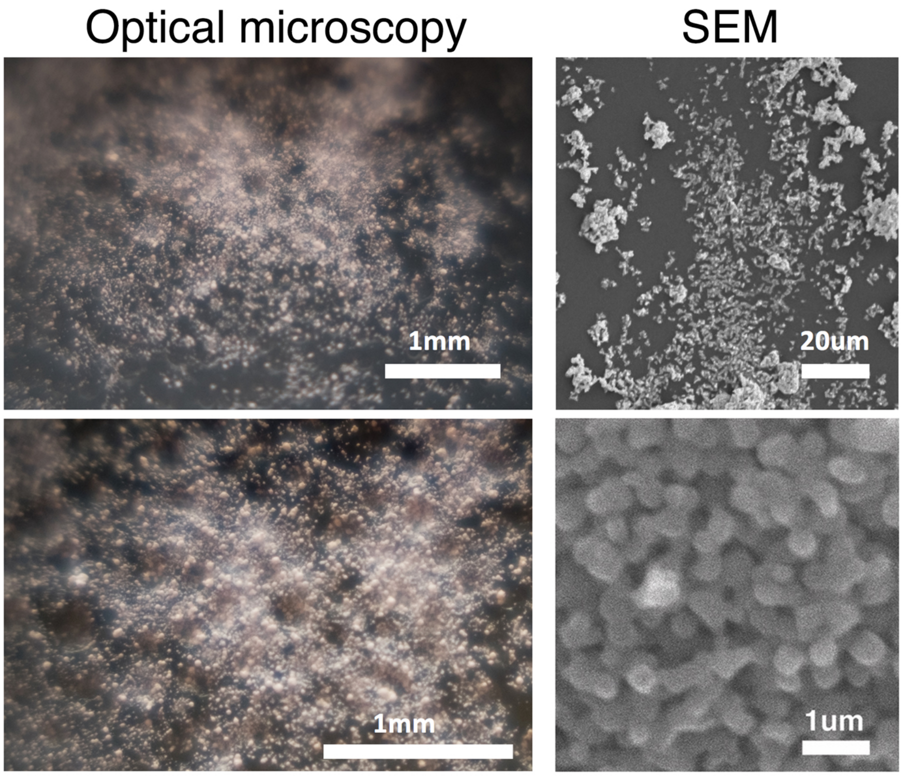

Supplementary Figure 10: Optical microscopy and SEM images of the BTO sample. The images show the uniform distribution of particles in nanopowder which ensures that if single Bragg reflection is detected on X-ray detector, during coherent diffraction imaging experiment, it corresponds to a single nanoparticle.

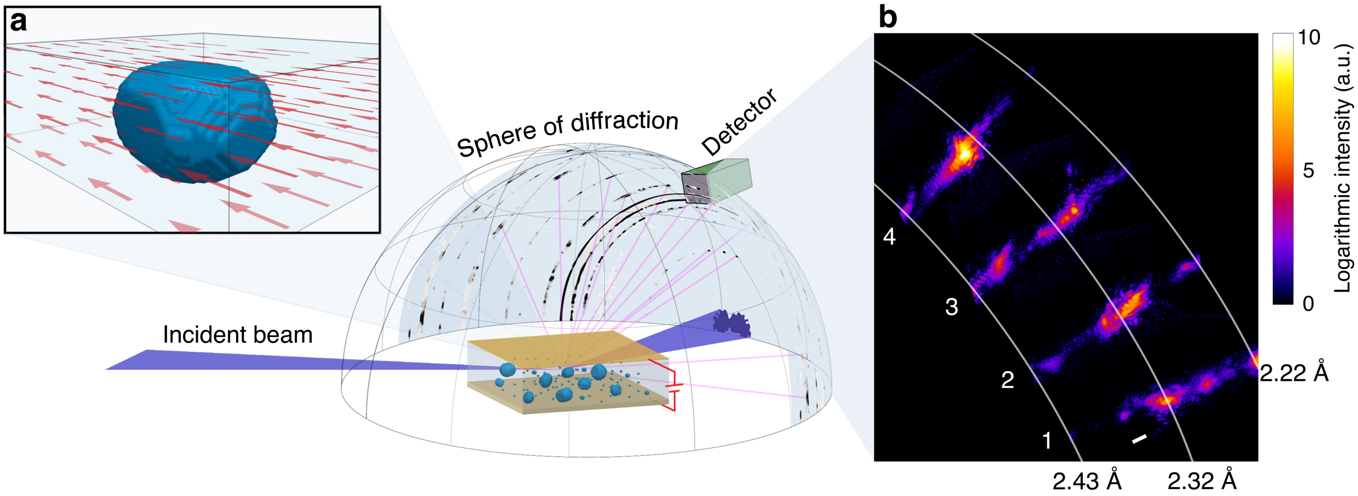

Supplementary Figure 11: Principle scheme of the BCDI experiment. a, Illustration of the experimental scheme with incident coherent X-ray beam being scattered from the extremities of the nanoparticle under applied electric field. Detector is used to record the diffraction patterns and is mounted on motorized arm to properly position the detector around the diffraction sphere for fine tuning into specific peak. **b,** Diffraction patterns recorded at different applied electric fields with numbering indicating evolution of the pattern from (1) for 0V of applied field, (2) 10V of applied field, (3) again 0V of applied field, and (4) for -10V of applied field. As given by phase field simulation, 10V corresponds to an electric field of 223 kV cm^{-1}across the single BTO nanoparticle. Scale bar corresponds to 0.1 $Å^{-1}$.

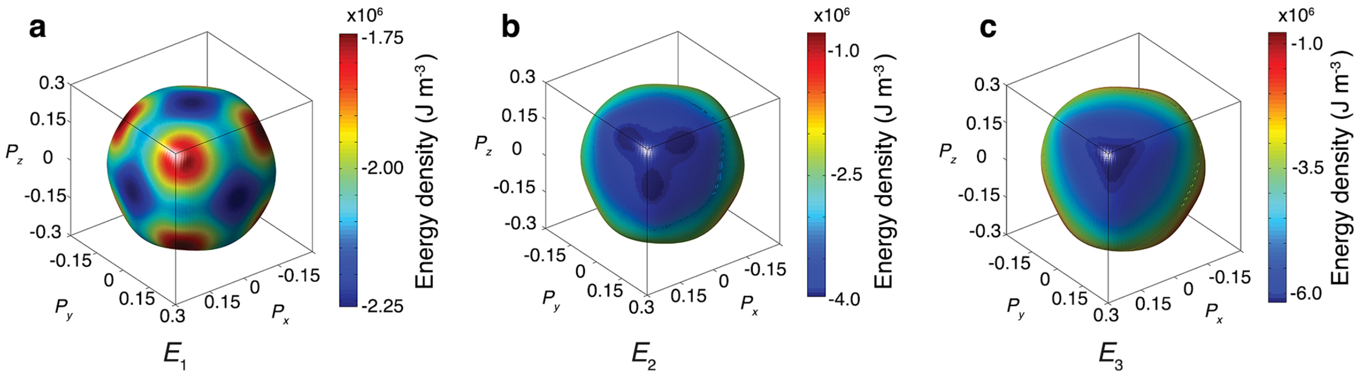

Supplementary Figure 12: Evolution of minimum free energy density of polarization P with the increasing of the applied electric field along (111) direction. a, Minimum free energy density at the initial zero field exhibiting primary T phase. **b,** The minima of free energy density is at M phase with the increasing of the electric field. **c,** Minimum free energy density at the maximum filed exhibiting primary R phase.

Supplementary Table 1: Elastic and electrostrictive coefficients of BTO used to fit polarization maps.

$c_{11}(10^{11}$ N·m$^{-2})$	1.78
$c_{12}(10^{11}$ N·m$^{-2})$	0.964
$c_{44}(10^{11}$ N·m$^{-2})$	1.22
Q_{11}(C^{-2}·m^{4})	0.10, 0.11
Q_{12}(C^{-2}·m^{4})	-0.034, -0.045
Q_{44}(C^{-2}·m^{4})	0.029, 0.059

Supplementary References:

1. R. Henderson, J. Moffat, The difference Fourier technique in protein crystallography: errors and their treatment. *Acta Cryst. Sect. B* **27**, 1414-1420 (1971).
2. C. M. Wu, W. J. Chen, Y. Zheng, D. C. Ma, B. Wang, J. Y. Liu, C. H. Woo, Controllability of vortex domain structure in ferroelectric nanodot: fruitful domain patterns and transformation paths. *Scientific Reports* **4**, 3946 (2014).
3. I. I. Naumov, L. Bellaiche, H. Fu, Unusual phase transitions in ferroelectric nanodisks and nanorods. *Nature* **432**, 737-740 (2004).
4. G. Pilania, R. Ramprasad, Complex polarization ordering in $PbTiO_3$ nanowires: a first-principles computational study. *Phys. Rev. B* **82**, 155442 (2010).
5. S. Prosandeev, I. Ponomareva, I. Naumov, I. Kornev, L. Bellaiche, Original properties of dipole vortices in zero-dimensional ferroelectrics. *J. Phys.: Condens. Matter* **20**, 193201 (2008).
6. S. Prosandeev, I. Ponomareva, I. Kornev, I. Naumov, L. Bellaiche, Controlling toroidal moment by means of an inhomogeneous static field: an ab initio study. *Phys. Rev. Lett.* **96**, 237601 (2006).
7. J. Slutsker, A. Artemev, A. Roytburd, Phase-field modeling of domain structure of confined nanoferroelectrics. *Phys. Rev. Lett.* **100**, 087602 (2008).
8. U. Pietsch, V. Holy, T. Baumbach, *High-resolution X-ray scattering: from thin films to lateral nanostructures* (Springer, 2013).
